# “At 150 kg, you can't run” men's weight loss stories in a popular health magazine provide appropriate examples of good health practice

**DOI:** 10.1080/21642850.2014.891947

**Published:** 2014-03-06

**Authors:** Danielle Couch, Gil-Soo Han, Priscilla Robinson, Paul Komesaroff

**Affiliations:** ^a^ The Monash Centre for the Study of Ethics in Medicine and Society, Monash University, The Alfred Centre Level 6, 99 Commercial Road, Melbourne, Victoria3004, Australia; ^b^ School of Media, Film and Journalism, Monash University, Clayton, Victoria3800, Australia; ^c^ School of Public Health and Human Biosciences, La Trobe University, Bundoora, Victoria3086, Australia

**Keywords:** obesity and weight management, media, weight loss, men's health, narrative

## Abstract

We explore weight loss stories from 47 men collected from the Australian edition of *Men's Health* magazine between January 2009 and December 2012. Our analysis uses a mixed methods approach that combines thematic analysis and descriptive statistics to examine weight loss strategies against clinical practice guidelines for the management of overweight and obesity. All the stories reported the use of physical activity for weight loss and most stories detailed dietary changes for weight loss. Our findings indicate that most of the men reportedly used some form of behavioural strategies to assist them in their behaviour change efforts. The weight loss methods used were consistent with clinical practice guidelines, with the exception of some dietary practices. As narratives may assist with behaviour change, stories like those examined in this study could prove to be very useful in promoting weight loss to men.

## 1. Introduction

### 1.1. Obesity

The “obesity epidemic” is of public health concern in Australia and globally. The World Health Organization (WHO) has indicated that in 2005, there were approximately 1.6 billion overweight adults (aged 15 years and older) and 400 million obese adults worldwide (WHO, 2011). In Australia, in 2011–2012, 63.4% of the population aged 18 years and over were overweight or obese (ABS, 2012). Approximately 70% of Australian men are overweight or obese, compared to 56.2% of Australian women being overweight or obese (Australian Bureau of Statistics, 2012). Body Mass Index (BMI) is a common population measure of obesity. A BMI of 25 kg/m^2^ or more is classified as overweight and a BMI of 30 or more is classified as obese (WHO, 2011).^1^ Classification for BMI scores and the level of associated health risk (risk of co-morbidities) are detailed in Table 1 (WHO, 2000).

**Table 1.** BMI classifications.

Classification	BMI (kg/m^2^)	Risk of co-morbidities
Underweight	<18.50	Low (but increased risk of other clinical problems)
Normal range	18.50–24.99	Average
Overweight:	>25.00	
Pre-obese	25.00–29.99	Increased
Obese class 1	30.00–34.99	Moderate
Obese class 2	35.00–39.99	Severe
Obese class 3	>40.00	Very severe

Various factors can affect weight gain or loss. At an individual behavioural level, excess weight in individuals is usually the result of an extended period of energy imbalance (National Health and Medical Research Council [NHMRC], 2013). Central to this imbalance are diet and physical activity (Kegler, Escoffery, Alcantara, Ballard, & Glanz, 2008; NHMRC, 2013), but these are influenced directly and indirectly by a broad range of behavioural, genetic, physiological, social, cultural and environmental factors, along with life stage (NHMRC, 2013). At an individual level, potentially effective weight loss interventions include: exercise, diet, behavioural strategies, a combination of exercise, diet and behavioural strategies, pharmaceutical interventions combined with strategies to change lifestyle behaviours, and surgery for some morbidly obese people (Nammi, Koka, Chinnala, & Boini, 2004; NHMRC, 2013; Orzano & Scott, 2004).

### 1.2. Health, weight and the media

Health and medical issues receive much coverage in the media, which are a key source of health information for many people (Chapman et al., 2009; Kirkman, 2001). It is recognised that the media cannot only influence public opinion (Chapman et al., 2009), but can also be a tool either to promote health or – through the promotion of unhealthy behaviours and lifestyles – to undermine health messages. The media can draw attention to specific health issues and problems, through news stories, advertisements or current affairs and fictional programmes. Media coverage and representations of health and illness create and re-create meanings of health and illness for the public and professionals (Lyons, 2000). Media analysis is, therefore, vital to the study of health.

Over the past few decades television, magazines and newspapers (Lyons, 2000), and more recently the Internet (Cotten & Gupta, 2004) have become increasingly important sources of health information. In addition to these, in the West, much health promotion and education occurs through popular printed materials (Daly, Kellehear, & Gliksman, 1997), and public health social marketing campaigns aimed at behaviour change often use a wide range of mass media to deliver their messages (Evans, 2006). Printed magazines have led the way in offering familiar, everyday, friendly lifestyle advice. In the latter part of the twentieth century increased coverage of the benefits of diet and exercise (Leonhard-Spark, 1978) became a feature of men's magazines, along with advice on bodily appearance and maintenance (Neumark Sztainer, 2005). In addition to this more general bodily coverage, men's weight loss success stories are becoming increasingly common in the popular media. Major weight loss companies, such as Weight Watchers and Jenny Craig, have also developed men's specific weight loss programmes, with associated “success stories” as part of their marketing strategies.

The promotion of commercial weight loss programmes and weight loss reality television have been criticised for being unrealistic, misleading and potentially dangerous (Blaszkiewicz, 2009; Freedhoff & Sharma, 2009). It has been suggested that success stories make weight loss seem easy and simple, and that they seldom discuss the weight gain relapses that are common in real life (Bonfiglioli, Smith, King, Chapman, & Holding, 2007). Additionally, weight loss success stories may also contribute to weight stigma by confirming “overweight people's misery by dissociating themselves with contempt and disgust from their former self” (Sandberg, 2007, p. 462). Advertisements for weight loss products and services have also been found to commonly present false and grossly exaggerated claims about performance and outcomes (Cleland, Gross, Koss, Daynard, & Muoio, 2002).

In addition to these criticisms, it has been argued that media representations of overweight, obesity and weight loss are subject to gender biases. On the one hand, overweight is presented predominantly as a women's problem. On the other, the reasons for attempting to lose weight appear to be different between men and women: specifically, women lose weight because of the way they look and men lose weight because of their health, and women's lives are transformed by their weight loss while for men life goes on as usual (Sandberg, 2007). Some research has suggested that men's core motivation to lose weight is to be more effective in their workplaces (Sabinsky, Toft, Raben, & Holm, 2007).

Men's lifestyle magazines, and their coverage of diet, exercise, body maintenance and appearance, have recently attracted much research, particularly in the fields of masculinities and gender identity (Alexander, 2003; Attwood, 2005; Benwell, 2004; Boni, 2002; Hall & Gough, 2011; Ricciardelli, Clow, & White, 2010), sexuality (Rogers, 2005; Taylor, 2005), body image and muscularity (Labre, 2005), and the social construction of health and masculinity (Bunton & Crawshaw, 2002; Crawshaw, 2007; Stibbe, 2012). To date, however, there has been little research into how weight loss specifically is presented in these magazines, including the strategies that these magazines promote and recommend.

In view of the problematic nature of much weight loss information in the media, along with the high rates of overweight and obesity in men, there is a need for improved strategies for addressing these issues, such as public health media advocacy and gender-relevant programmes. An examination of men's weight loss stories might contribute to a better understanding of the weight loss advice and messages that are being promoted to the men reading these magazines, and of how they compare to previous findings about weight loss in the media. It may also provide insights into the usefulness of narratives for addressing broader public health issues and inform new public health media advocacy strategies.

In this study, we have explored men's weight loss stories published in the popular men's magazine, *Men's Health*. We have examined the weight loss strategies used and the time taken for weight loss, as described in these stories, and considered the appropriateness of this information in the light of clinical practice guidelines^2^ and current evidence for overweight and obesity management, along with the wider theoretical literature. We have also considered how our findings may contribute to the development of novel communication strategies for addressing other public health problems.

## 2. Methodology

*Men's Health* is a monthly men's lifestyle magazine in Australia, although local editions are also published in many other countries. According to readership estimates, this publication is the most widely read men's lifestyle magazine in Australia (Roy Morgan Research, 2009, 2010, 2011, 2012), with an estimated 404,000 readers in June 2012 (Roy Morgan Research, 2012). Each edition of this magazine publishes a man's weight loss story, under the general banner title “Gutless Wonders”.^3^ These stories comprised the sources of data for this study. Sampling commenced in January 2009 and continued through until December 2012, during which time a total of 47 weight loss stories appeared. We collected complete years to account for any seasonal variations that might have affected the content. Data collection was ceased after four years as the analysis reached saturation: that is, as the stories ceased to provide “new” information (Strauss & Corbin, 1998). A copy of each weight loss story was made and imported into NVivo9, a qualitative analysis software program, for storage and analysis.

Our sampling from *Men's Health* magazine followed a purposive approach using three main criteria: genre, popularity and gender of readership. Specifically, we had set out to identify stories concerning obese men who had lost weight appearing in a high-circulation Australian men's magazine. It should also be noted that these stories also appeared on the *Men's Health* Australia website, suggesting an even wider reach and readership than is suggested by the magazine readership estimates.

We used a mixed methods approach to analyse the data, which included both thematic analysis and descriptive statistics. The textual data were analysed in relation to thematic categories, in order to obtain a detailed, rich and complex account of the main ideas and concerns (Braun & Clarke, 2006). We specifically sought to undertake an inductive interpretation of data and to review them in relation to extant theoretical constructions (Liamputtong, 2009), thereby incorporating sequentially both “inductive” and “theoretical” thematic analysis (Braun & Clarke, 2006). While it is important to note that no research is conducted in a theoretical or epistemological vacuum, in the inductive thematic analysis phase the data coding process was approached without trying to apply any pre-existing coding frame or theory – the coding and analysis was data-driven or “grounded”. In the theoretical thematic analysis phase, the coding and analysis were driven by the researchers' “theoretical or analytic interest in the area … thus more explicitly analyst-driven” (Braun & Clarke, 2006, p. 12). While both phases were important, the latter provided a focus for particular insights into the potential public health impacts of men's weight loss stories, and for responses to the problem of obesity as a health issue.

We commenced by reading the articles and noting initial ideas and thoughts. We undertook further reading and re-reading, reviewing and open-coding the articles (Braun & Clarke, 2006). Next, we identified themes in the codes, grouping certain codes together, and then further reviewed and refined these themes (Braun & Clarke, 2006). From our knowledge of clinical and public health weight management strategies, the emergent themes and categories from our inductive thematic analysis were clearly in line with clinical and public health strategies for weight management. After identifying this, we refined our themes and then undertook further reading and coding of the data explicitly informed by this knowledge; this was our theoretical thematic analysis. We then further reviewed and considered the identified themes against clinical guidelines and relevant theory and evidence.

Additionally, we undertook a small amount of quantitative analysis. Each weight loss story included a small table of information on the individual, which included his age, height before and after weight loss. From this information, we calculated BMIs and then categorised the data according to the classification for BMI scores and the associated health risks (as per Table 1). Many of the weight loss stories provided an indication of the length of time taken for weight loss, this information generally being provided with a start and end month and year. Using these data, we were able to estimate the time for weight loss to occur and the mean amounts of weight loss per month.

## 3. Findings

We provide a summary of the individual demographic and weight data presented in the weight loss stories and then present the findings according to the main themes which emerged from our inductive and theoretical thematic analysis, as follows: physical activity; eating strategies; behavioural strategies; and time taken to lose weight.

### 3.1. Overview of data

With the exception of October 2010, each issue of *Men's Health* (Australia) magazine examined included a weight loss success story, so that in total we collected 47 weight loss stories. The age of the men in these stories ranged from 20 to 43 years, with a mean age of 28.6 years. Before and after BMIs for each individual, the range and mean BMIs before and after and the mean percentages of body weight lost are presented in Table 2.

**Table 2.** Participant demographic details.

Demographic category	*N* (47)
*Age* (*years*)	
Mean	28.6
Range	20–43
*Weight* (*kg*) *– before*	
Mean	128.55
Range	90–185
*Weight* (*kg*) *– after*	
Mean	83.30
Range	64–103
*Weight loss* (*kg*)	
Mean	45.15
Range	15–91
*BMI – before*	
Mean	39.11
Range	29.70–57.1
Obese class 3	14
Obese class 2	22
Obese class 1	10
Pre-obese	1
*BMI – after*	
Mean	25.35
Range	22.07–30.46
Obese class 1	2
Pre-obese	22
Normal	23
*% of body weight lost*	
Mean	34.17
Range	15.00–49.34

BMI classification changes are presented in Table 3.

**Table 3.** BMI classification changes.

BMI category changes	N
Obese class 3 to obese class 1	2
Obese class 3 to pre-obese	9
Obese class 3 to normal	3
Obese class 2 to pre-obese	11
Obese class 2 to normal	11
Obese class 1 to pre-obese	2
Obese class 1 to normal	8
Pre-obese to normal	1

As indicated in Table 3, at the start of their weight loss journeys none of the men were in the “normal” BMI category, whereas 23 men completed their weight loss story with a normal BMI. Twenty-two of the men were categorised as pre-obese at the end of their weight loss story; of these nine had previously been obese class 3, eleven had been obese class 2 and two had been obese class 1. Two men were classified as obese class 1 at the end of their weight loss journeys, both having been classified as obese class 3 before they began their weight loss.

### 3.2. Physical activity

Every story included information on the role of physical activity in achieving weight loss. Most (46) of the stories explained that the men undertook cardiovascular exercise to assist in their weight loss, such as walking, cycling, skipping, using a rowing machine, swimming, running and playing team sports. Many (34) experienced significant progress in their cardiovascular exercise. For example, some (12) started with walking and then advanced to running: “One kilometre became 2 km, which became 3 km, and eventually I was easily running 5 km several times a week” (story #16, aged 28). Another who also started walking before running stated: “at 150 kg, you can't run” (story #23, aged 34). A few (8) of the men progressed to competing in events such as triathlons and boxing competitions.

Just over half of the stories included details of some form of resistance training to assist with weight loss, including weight training and body weight exercises. As one man noted: “If I could do it all again, I'd do weights from the beginning. It speeds up your metabolism and helps give you definition” (story #3, aged 24). As this comment suggests, a few stories also noted the importance of resistance training to build muscle for aesthetic reasons. The stories of the men who undertook resistance training also demonstrated progression in their resistance training, in the weight they could lift and the frequency and intensity of this training.

All the stories provided information on how the men undertook regular physical activity, generally providing details of the duration per day and number of times per week. All the men reportedly exercised several times each week. For example, “Four days a week he combines weight training with 30 minutes of intense cardio” (story #17, aged 23) and “ran at the gym for 45 minutes 4–5 times a week” (story #1, aged 33). A few (4) of the men's stories noted how they also built physical activity into their day-to-day activities, such as using walking or cycling as a mode of transport to reach destinations. Four men enlisted the help of personal trainers.

### 3.3. Eating strategies

Most (41, or 81%) of the men's weight loss stories included some details of how they modified their eating to assist with weight loss. This included portion control, “healthy” eating, reduced alcohol consumption, special diets and expert advice.

A few (9) of the men's stories noted that reduction of the portion sizes in their meals was important. A few (11) of the stories also noted that the men increased the frequency with which they ate: for example, one man would “eat small meals every two hours” (story #31, aged 27). Only two of the stories explicitly referred to counting kilojoules, with one using an iPhone app and the other “employing a mathematical approach to counting kilojoules and fat” (story #1, aged 33). Many (28) of the men who reportedly changed their eating for weight loss described “healthy” eating behaviours in which they reduced or eliminated junk food, soft drinks and takeaway and increased lean meats, grains and fruit and vegetables, along with cooking for themselves. One man noted, “[I] made sure that I got the right balance of carbs [*sic*], protein and vegetables” (story #8, aged 25). A few (7) others noted that they were successful in changing their eating behaviours by making small and gradual changes, and food substitutions, such as skim milk for full cream milk, or another man who swapped “milk for water in his protein shakes and substitute[d] bread for flat wraps with his sandwiches” (story #41, aged 25).

A few (6) men's stories noted that they allowed themselves a day each week or fortnight to eat whatever they felt like as “You've got to have those days or you'll go batty” (story #37, aged 27). Another explained that he avoided thinking about his new eating behaviours as a diet: “The word ‘diet’ is toxic. You have to allow yourself the odd piece of cake or slice of pizza on special occasions” (story #35, aged 24).

Reduced alcohol consumption was also important for a few (11) men. These men reported that they reduced both the amount they drank in a session and the frequency of these sessions. One man's story indicated that his reduced drinking had a spillover effect as it also helped to improve his eating behaviours, because for him drinking alcohol and eating junk food were often combined activities. In addition to the changes in eating and drinking behaviours, four of the men who were smokers also reportedly gave up smoking during their weight loss efforts.

Special diets of some form were present in some (20) stories, even for those who described more general “healthy” eating behaviours. Special dietary changes included a “home detox”, reducing “carbs” or only eating “carbs” at certain times of the day, with a few men's stories specifically noting they followed a high protein/low carb diet. One man's controlled carbohydrate intake was outlined as “His only carb fix was his oatmeal in the morning” (story #31, aged 27). Two men used commercial shakes for meal replacements. Three sought professional advice to help them change their eating behaviours, two from dieticians and one from a doctor.

### 3.4. Behavioural strategies

Most (42, or 89%) of the weight loss stories detailed some kind of behavioural strategy to help with weight loss efforts. Some (24) of the men's stories highlighted the importance of goal setting. These goals included training for a sporting event, aiming for a certain clothes size or working towards a certain time frame. One man “booked a holiday towards the end of the year, creating a deadline to aim for” (story # 19, aged 26) and for another:

After five months he'd lost just over 20 kg and found himself under 100 kg for the first time in 19 years. Wary of the temptation to slack off, he decided to train for a triathlon, completing his first sprint event. (story #43, aged 35)

In contrast with these longer-term goals, one man noted the importance of setting reasonable and achievable goals: “Set realistic goals. If you set small, monthly or weekly targets, you have a better chance of hitting them, which helps you sustain motivation” (story #31, aged 27).

Some (19) of the men reported that support from friends and family and work mates provided motivation for them to keep up their weight loss efforts. These men stated that positive comments about their weight loss efforts and encouragement to keep going were very helpful. One man stated: “My kids, friends and workmates were incredibly supportive – that really helped keep me on track and I haven't looked back” (story #6, aged 43). A few (5) men used a desire to be active with their children as a motivator to help in their weight loss: “I wanted to be able to run around with my son and have fun with him” (story #5, aged 28).

Many (31) of stories indicated that the men also undertook some method of monitoring or tracking of their physical activity, eating behaviours and/or weight loss, which reportedly helped them to achieve their weight loss. For example, one man noted how he weighed himself and wrote the weight at the top of a blank sheet of paper that he left beside the bathroom scales: “I would then weigh myself every single morning and track my progress” (story #11, aged 24). A few (4) men's stories noted how they kept food diaries to help them monitor their diets. The monitoring of the amount of physical activity each undertook, as detailed previously, is another example of the measuring and tracking of progress.

### 3.5. Time taken to lose weight

Thirty-nine (or 83%) of the weight loss stories provided an indication of the length of time taken for their weight loss. The shortest duration of time for weight loss in the 47 stories was three months for an individual who lost 15 kg; this was also the smallest amount of weight lost across all of the stories. The longest duration of time for weight loss was approximately 72 months for an individual who lost 69  kg. The greatest amount of weight lost was 91  kg, although the time taken for this weight loss is unclear as one part of the story states the weight was lost within 9 months, yet other information in the story suggests a period of approximately 20 months. These discrepancies impact on further calculations for all the stories, so presented results use both possible weight loss durations. Using the 9 months period, the mean amount of weight loss per month was 3  kg, and the range of weight loss per month was 0.45–10.11  kg. Using the 20 months period, the mean amount of weight loss per month was 2.9  kg, and the range of weight loss per month was 0.45–6.2  kg. A few of the weight loss stories outlined how the individuals' weight losses were not linear; that is, they lost some weight, they then gained some weight (due to personal, romance or motivation issues) and then they re-focused on their efforts and lost weight again. All the stories indicated that the men lost weight gradually over time.

## 4. Discussion

In this section, we review our findings and consider the strengths and limitations of our study. Our use of mixed methods (descriptive statistics and inductive and theoretical analysis) has allowed us to consider our findings against standards of practice (e.g. NHMRC guidelines, 2013) and the wider theoretical literature.

Most of the men featured in the stories analysed employed physical activity, dietary changes and behavioural strategies to help with weight loss. This is consistent with evidence which suggests that multi-component interventions that address physical activity, nutrition and behaviour change are most effective (Kirk, Penney, McHugh, & Sharma, 2012). In the following sections, we discuss how the men used these different strategies.

### 4.1. Overview of data

All of the men lost large amounts of weight, with the mean weight loss being 45.2  kg. Given that even small amounts of weight loss can result in improved health (NHMRC, 2013), the weight loss described in these stories is suggestive of health improvements in all the men. Two men were classified as obese class 1 at the end of their weight loss journeys and 22 were categorised as pre-obese. Many were undertaking resistance training, so their BMIs may not be fully reflective of changes to their lean muscle mass, as people with high muscle mass may have less adipose tissue than less muscular people, so that a higher threshold BMI might be appropriate for these men (NHMRC, 2013). Ethnicity, which we did not examine as it was not reported in the stories, can also affect BMI (WHO, 2000): for example, Pacific Islander populations tend to have a higher proportion of lean body mass (NHMRC, 2013), and it is possible that this may have been relevant to some of the men in the stories.

The men lost an average of 34.2% of their “initial” body weight. As many people regain weight after weight loss, it has been suggested that a realistic amount of longer-term weight loss is around 5–10% of the starting weight (Anderson, Vichitbandra, Qian, & Kryscio, 1999; NHMRC, 2013), but when diet and exercise are combined, weight loss is greater at one year compared with diet alone as the weight loss method (Curioni & Lourenco, 2005). Most of the men in the stories did combine diet and exercise. As our data present only a snapshot in time for the men in the stories, it is not possible to determine if they sustained their large weight losses over time. However, exercise was integral to all the men's weight loss and if this was continued the amount of weight regained over time may well have been limited.

### 4.2. Physical activity

All of the men in the stories undertook physical activity, with nearly all undertaking cardiovascular activity and many also resistance training. Recent evidence suggests that the relationship between physical activity and health benefits is direct and curvilinear, with the greatest benefit occurring in those who start from a baseline of no physical activity. For people who are inactive, even small increases in the physical activity they undertake provide important health gains (Powell, Paluch, & Blair, 2011). Given that most of the men were not exercising at the commencement of their weight loss efforts, it is likely that they received great health benefit from their exercise efforts.

Frequent physical activity is more beneficial than infrequent (i.e. one or two days per week) physical activity (NHMRC, 2013), and increases in physical activity should be made gradually to reduce the likelihood of adverse events, such as injuries, and to improve adherence (Powell et al., 2011). The men in the stories all gradually increased their physical activity frequency and intensity, to the point where their routines were consistent with current evidence-based recommendations (NHMRC, 2013). It is also important to consider what type of physical activity is appropriate for obese people as the degree of “obesity, fitness level, comorbidities and age” may all impact on what is appropriate (NHMRC, 2013, p. 43). The men in our stories who started walking because they were too large to run are examples of starting with appropriate physical activity. Increases in incidental activity are also recommended for weight management and health (NHMRC, 2013); the men who made efforts to walk or cycle to destinations as a way of building physical activity into their regular daily activities illustrate how incidental activity can be used in weight loss efforts.

Approximately, half of the men in the stories undertook some form of resistance training to aid their weight loss efforts. As muscle-strengthening activities, such as resistance training, offer important benefits such as improvements to metabolic and musculoskeletal health (NHMRC, 2013), they were again participating in beneficial physical activities. The fact that some undertook resistance training to build and shape muscle for aesthetic reasons suggests that some men may wish to lose weight for other than health reasons, for example, to achieve a body ideal and not just a weight outcome.

The regular physical activity detailed in stories of men might be useful in maintaining their weight loss if they continued engaging in high levels (about one hour per day) of physical activity, as this has been found to be associated with successful long-term weight loss maintenance (Hill, Wyatt, Phelan, & Wing, 2005; Wing & Phelan, 2005).

The physical activity undertaken for weight loss by the men in the stories is generally consistent with evidence-based recommendations, but it is important for health professionals to recognise that weight loss efforts may not be motivated solely by health concerns. Thus, public health obesity campaigns that focus on health issues as part of the core message may not be reaching those who are more interested in weight loss for aesthetic reasons.

### 4.3. Eating strategies

A routine that will produce a 2500 kJ per day energy deficit is recommended for overweight or obese adults (NHMRC, 2013). While most of the men in the stories modified their eating behaviours to assist in weight loss, only two specially monitored their kilojoule intake, but neither of these stories describing this provided specific details of the kilojoules deficit per day for which these men were aiming; as such a comparison cannot be made against the recommended deficit of 2500 kJ per day.

A few (9) of the men in the stories employed portion control as a method in their weight loss efforts. A few (11) also increased the frequency of their smaller meals in an effort to keep their metabolic rates higher. Food portion control or reduction is recommended to assist with weight loss (NHMRC, 2013) and, while evidence regarding the benefits of more frequent meals for weight loss and weight management is inconsistent (Bachman, Phelan, Wing, & Raynor, 2011; Bachman & Raynor, 2012; Cameron, Cyr, & Doucet, 2010; Leidy, Tang, Armstrong, Martin, & Campbell, 2011; Ohkawara, Cornier, Kohrt, & Melanson, 2013; Palmer, Capra, & Baines, 2011), this often appears as a recommended method in popular media. For example, *Men's Health* magazine has published articles promoting the benefits of frequent meals and snacks. One article encouraged four meals per day and snacks. It included a second breakfast because:

eating twice in the morning means you'll take in fewer kilojoules overall”, it then encourages snacks throughout the day as “You can't lose weight and keep it off unless you snack! In fact, studies suggest that people who eat less often than three times a day may have trouble controlling their appetite. (Australian Men's Health, 2013)

For many people the reality of science is what they learn from the media (Conrad, 1997). The men advocating the benefits of eating more meals more frequently to aid their weight loss efforts may be applying what they believe is an effective, research based, weight loss strategy as promoted in the popular media. Yet the media has been identified as disseminating misinformation based on inadequate reporting where scientific sources are were “uncritically reiterated and amplified” (Michelle, 2006, p. 55) – in this instance, the mixed evidence on meal frequency is being disregarded or ignored, and one set of findings are being promoted. It has been suggested that such reporting creates “cultural residues”, whereby obsolete, or incorrect, ideas become part of public knowledge and discourse (Conrad, 1997, p. 149). The men's stories with details of frequent meals as a weight loss strategy, along with supporting editorial copy as exemplified above, are likely to be contributing to supporting weight loss practices for which the evidence is unclear. The men's stories which noted reduction in alcohol intake are consistent with the recommendation that doctors should encourage alcohol intake reduction in people trying to lose weight as alcohol may contribute to fat storage (NHMRC, 2013). The men who undertook food substitutions as a method of changing their diet are also in line with recommendations to make small changes to eating habits that can assist in sustaining such changes (NHMRC, 2013).

The “special” diets that some men undertook, such as those high in protein and low in carbohydrates, meal replacement shakes and very frequent meals, may be indicative of muscularity-oriented disordered eating behaviours (Griffiths, Murray, & Touyz, 2012). That is, such eating patterns may be driven by a desire to achieve a muscular body ideal. All the stories discussed physical activity, but not all discussed eating strategies for weight loss (41 of 47 included eating strategies). This may be because dieting is more commonly seen as part of the female domain (Gough, 2007), but it may also suggest there is opportunity for public health media interventions further promoting the benefits of healthy eating specifically to men.

While some of the eating behaviours detailed in the men's stories are in line with evidence-based recommendations, a number of the eating and dietary strategies, such as small, frequent meals and the “special” diets present in the stories, are not based on evidence and may be even considered risky if they are related to disordered eating behaviours.

### 4.4. Behavioural strategies

Most of the men in these stories employed behavioural techniques that could be considered effective self-management strategies. Evidence-based practical advice for self-management as part of a multi-component intervention (e.g. physical activity and dietary changes) includes short-term goal setting (Pettman et al., 2008), self-monitoring of behaviour and progress through keeping a food and/or exercise diary, involvement of family and friends where/if appropriate, regularly self-weighing and rewarding oneself for reaching a goal (NHMRC, 2013).

Some (24) of the men in the weight loss stories detailed how they set goals to help them achieve weight loss, and some noted the importance of support from family and friends to keep them motivated in their weight loss efforts. Many (31) of the men undertook some kind of monitoring and tracking of their eating, such as food and exercise diaries and daily weigh-ins with recording of weight. A few rewarded themselves with days where they could eat whatever they liked, such as pizza or cake, to help keep them motivated.

In general, the stories demonstrated a range of self-management and behavioural strategies that are similar to those recommended in clinical practice guidelines for the management of overweight and obesity (NHMRC, 2013).

### 4.5. Time and effort to lose weight

Nearly all the stories indicated that the men's weight loss was fairly slow and steady. This is in contrast with other research examining the presentation of weight loss stories in the media, where weight loss was presented as occurring very quickly and in an unsustainable manner (Boulos, Vikre, Oppenheimer, Chang, & Kanarek, 2012; Thomas, Hyde, & Komesaroff, 2007). Weight loss advertisements in particular have been criticised for their emphasis on rapid weight loss and on omitting reference to the need for diet and exercise (Cleland et al., 2002). It is noteworthy that the weight loss presented in the men's stories was slow despite *Men's Health* magazine being a channel for the advertisements of many body-weight-related products and advertisers can influence editorial (Rinallo & Basuroy, 2009).

The story in which we identified an improbable rate of weight loss, amounting to 91  kg over 9 months, would have been somewhat more believable if the weight loss was spread across 20 months. This discrepancy highlights the mediated nature of the stories and suggests that some “poetic” or narrative license might be used in these stories, or it may have been a copy error.

Previous research has suggested that the media often portray weight loss as easy and simple (Bonfiglioli et al., 2007); however, in the stories we examined the amount, type and progression of physical activity, along with changes to eating behaviours, psychological motivators used to aid in weight loss, and the time taken to lose weight are not suggestive of weight loss as easy, quick or simple. A few stories also discussed how weight loss was not linear; in these stories, the men detailed how they had lost weight for a time but then regained weight due to issues in their life or challenges with ongoing motivation. Examples of struggles such as these might resonate with readers, who themselves have attempted to lose weight, succeeded for a while, but regained weight due to other issues. The stories under examination detailed the significant efforts and hard work each man undertook to lose weight, over an extended period of time.

### 4.6. Narratives, health and behaviour change

A narrative may be described as “any cohesive and coherent story with an identifiable beginning, middle, and end that provides information about scene, characters, and conflict; raises unanswered questions or unresolved conflict; and provides resolution” and can include historical accounts, personal experiences, good stories, the experiences of others and faith and religion (Hinyard & Kreuter, 2007, p. 778). The men's weight loss stories in our study may be considered narratives as they tell a coherent personal story of each man's weight loss journey.

Narratives “in the forms of storytelling, testimonials, and entertainment” have been shown to improve health behaviours in multiple settings (Meisel & Karlawish, 2011, p. 2022). They may assist in behaviour change by: (i) facilitating observational learning; (ii) overcoming resistance to a message by reducing counter arguments; and (iii) enabling identification with characters in a narrative and influencing the perceptions of personal and/or group susceptibility as well as social norms (Hinyard & Kreuter, 2007).

Much of the information about obesity and weight management presented in public health campaigns is based on epidemiological data about health risks and behaviours, whereas the men's weight loss stories in *Men's Health* magazine may offer readers an advantageous alternative to encourage weight loss. These stories may assist men in their weight loss efforts by providing opportunities for learning about successful methods of weight loss used by others, by offering opportunities to identify with the men in the stories as they work through personal struggles and triumphs, and through helping overcome resistance to change. The format used in *Men's Health* for these stories – monthly, one page only, focused on the journeys of individuals, and generally consistent with clinical guidelines and health recommendations – might be useful in addressing other health issues and could offer new directions for health behaviour change and innovative public health interventions. A recent Australian anti-gambling campaign using television advertisements, instead of editorial, has deployed a similar narrative format; the campaign provides brief narratives, showing issues experienced by individuals who are problem gamblers as they embark on their efforts to stop gambling. It has been commended for attracting high levels of public interest and engagement (Victorian Responsible Gambling Foundation, 2013). Narratives have also been found useful in cancer prevention and control (Kreuter et al., 2007).

### 4.7. Strengths and limitations of the study

The strength of our study is that we sampled stories for four consecutive years, ensuring that we accounted for any seasonal variation and that we were able to achieve data saturation. A further strength of our study was our use of mixed methods. By combining descriptive statistics with substantive inductive and theoretical thematic analysis, we have been able to develop and present a comprehensive understanding of the data. This has allowed us to consider our findings against clinical guidelines and the broader theoretical discourses around the media, weight and public health.

A limitation is that we only examined weight loss success stories, as these were the only weight management-related stories available in *Men's Health* magazine. An examination of stories about unsuccessful weight loss attempts may reveal different findings. The BMI data analysed were drawn from self-reported data which has been mediated by the magazine.

Additionally, we did not consider whether the men's weight loss stories were stigmatising. Stigmatising stereotypical and negative content about obese individuals is commonplace in the media (Ata & Thompson, 2010; Puhl & Heuer, 2009), and weight loss success stories can contribute to such negative outcomes (Sandberg, 2007). The weight loss stories analysed in this study could well play a role in supporting weight-related stigma, and so both produce an adverse impact on some readers and more broadly contribute to social norms around weight stigma. These stories might also discourage some from trying to lose weight as the hard work and long-term commitment demonstrated in most of the stories may seem overwhelming to some wanting to initiate weight loss efforts. Future studies could examine such stories to determine if they also contribute to weight stigma in the same way other obesity and weight loss media has been found to do (Puhl & Heuer, 2009), and if they provide motivation or act as a form of discouragement.

Additional research could examine men's responses to stories to determine if they in fact assist in behaviour change. Studies might also examine whether media-based narratives contribute to change in relation to other health issues. Research might also examine how audiences respond to weight loss stories which demonstrate slow and steady weight loss using physical activity, dietary changes and behaviour modification in comparison with weight loss stories which encourage unrealistic, rapid weight loss.

## 5. Conclusion

The men's stories examined in this study detailed weight loss efforts that included physical activity, dietary changes and behavioural modification as part of the strategies. Most of the latter were consistent with evidence-based recommendations. This is in contrast with most previous findings which indicate that media portrayals of weight loss as unrealistic and very rapid. The present study suggests that media portrayals of weight loss are more complex and nuanced than what has been previously suggested. By analysing men's weight loss success stories, we have demonstrated that there are alternative presentations of weight loss trajectories present in mainstream, popular media. Given the high rates of overweight and obesity in men media, stories of weight loss such as the ones examined in this study provide an additional avenue for health practitioners to consider in efforts to tackle obesity. The use of personal narratives can be useful, in addition to more common public health messages about the health risks of obesity. Media-based personal narratives that address public health issues may offer a new and innovative approach for engaging with health audiences.

## Notes

1. BMI is calculated as weight (in kilograms) divided by the square of height (in metres). The resulting score is then used to determine that person's weight classification and the health risks associated with this classification (WHO, 2000).

2. We have used Australian clinical practice guidelines for the management of overweight and obesity: *Clinical practice guidelines for the management of overweight and obesity in adults, adolescents and children in Australia* (NHMRC, 2013). The Australian guidelines were developed using the most recent systematically developed guideline for clinical management of overweight and obesity as a reference – the *2010 Scottish Intercollegiate Guideline Network (SIGN) Management of obesity: a national clinical guideline*. In addition to using the Scottish guidelines as reference guidelines, a systematic literature review was undertaken to examine areas relevant to Australian practice and areas which would benefit from an update of the literature. The recommendations within the Australian guidelines were then developed using NHMRC (2009) Levels of evidence and grades for recommendations for developers of guidelines and NHMRC (2011) Procedures and requirements for meeting the 2011 NHMRC standard for clinical practice guidelines. In the absence of quality evidence consensus-based recommendations were formulated (NHMRC, 2013).

3. Whilst we have used the Australian edition of *Men's Health* magazine for our data collection, editions from other countries include similar weight loss stories. For example the UK edition has *The Men's Health Fat Burners* and the US edition has the *Belly Off!* section.
